# Genetic Strategies to Detect Genes Involved in Alcoholism and Alcohol-Related Traits

**Published:** 2002

**Authors:** Danielle M. Dick, Tatiana Foroud

**Affiliations:** Danielle M. Dick, Ph.D., is a postdoctoral fellow, and Tatiana Foroud, Ph.D., is an associate professor, both in the Department of Medical and Molecular Genetics, Indiana University School of Medicine, Indianapolis, Indiana

**Keywords:** genetic theory of AODU (alcohol and other drug use), genetic linkage, genetic correlation analysis, genetic screening method, genome, genetic trait, QTL (quantitative trait locus) mapping, mutation, AOD dependence potential, alcoholic beverage, DNA

## Abstract

Researchers are using a variety of sophisticated approaches to identify genes that contribute to the development of alcoholism in humans or influence other alcohol-related traits. These strategies include linkage approaches, which can identify broad chromosomal regions that are likely to contain genes predisposing to the disorder, and association approaches, which test the association between a particular marker allele and a specific outcome. Animal studies using diverse strategies can also help identify genes or DNA regions that influence alcohol-related traits in humans. The results of these analyses are likely to have implications for fields such as genetic counseling, gene therapy, and pharmacogenetics.

Alcoholism is one of the most common and costly health problems in the United States. Substantial evidence from family, twin, and adoption studies suggests that genetic factors play a role both in normal patterns of alcohol use and in alcohol use disorders (i.e., alcohol abuse and dependence). It is estimated that approximately 50 to 60 percent of the variance in alcohol dependence can be attributed to genetic factors (McGue 1999). Researchers are currently attempting to identify the specific genes involved in patterns of alcohol use and alcohol dependence. These efforts are complicated by the complex nature of alcoholism and its development. Thus, although studies have convincingly demonstrated that genes play a role in the development of alcoholism, the same studies have also provided strong evidence for the importance of environmental factors. The genetic and environmental factors likely interact to result in disease development (for a more detailed discussion of those interactions, see the article in this issue by Heath and Nelson, pp. 193–201).

Despite these complexities, new developments in genetic technologies are enhancing scientists’ understanding of alcoholism. Several of these advances are described throughout this issue. This overview provides an introduction to some of the strategies currently being used to search for genes involved in alcoholism. It also discusses the implications of such basic genetic research for applied clinical practice.

## The Human Genome

**Figure 1:**
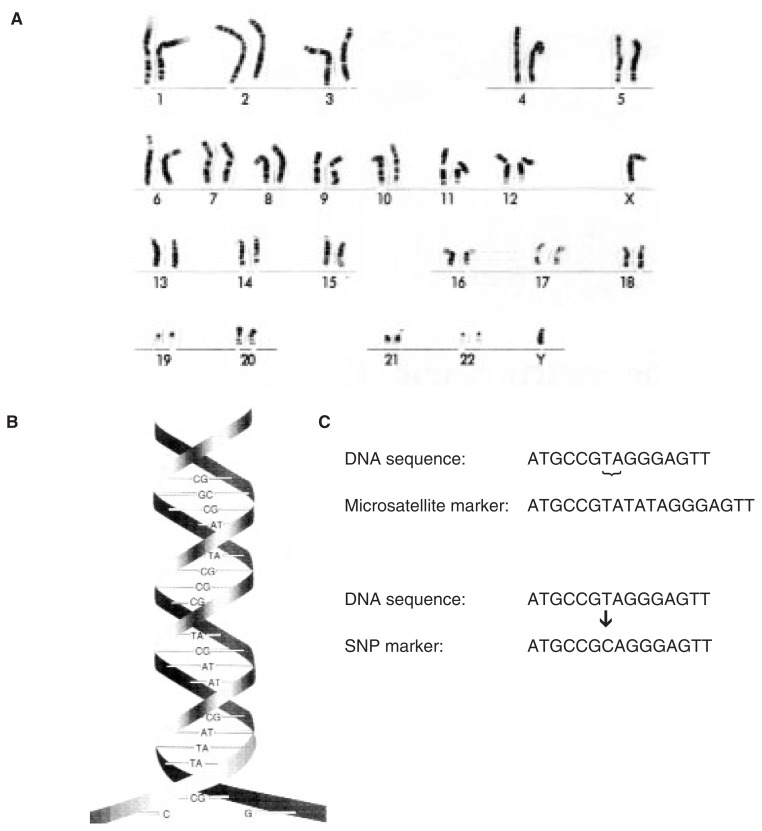
(A) A set of human chromosomes as seen under a microscope, containing 22 chromosome pairs (ordered according to size) and 2 sex chromosomes. In this case, the chromosomes were obtained from a male, as indicated by the presence of an X and a Y chromosome. (B) The structure of DNA. The DNA molecule is composed of two strands of building blocks that interact with each other. Each building block contains a chemical group called a base. There are four bases, adenine (A), cytosine (C), guanine (G), and thymine (T), in a sequence of paired bases. The base A on one strand always pairs with T on the opposite strand, and G always pairs with C. The sequence of these bases encodes the genetic information. (C) Micro-satellite and single-nucleotide polymorphism (SNP) markers. Microsatellite markers are short sequences of two to four bases (in this example, the bases T and A) that are repeated several times. The number of repeats differs among individuals, creating many different versions (i.e., alleles) of the marker for genetic analyses. For SNP markers, only a single base differs between individuals (in this case, the base T is changed to a C); thus, there are only two possible alleles of the SNP.

In most cells of the human body, the genetic information is contained in 46 microscopic structures in the nucleus, called the chromosomes. The first 22 chromosomes are present in pairs, and the 23rd pair consists of either 2 X chromosomes (female) or an X and Y chromosome (male) (see figure 1A). The chromosomes are inherited from the parents, with each parent providing 1 set of 23 chromosomes. These chromosomes contain a large molecule called deoxyribonucleic acid (DNA) (see figure 1B). The DNA consists of four building blocks called nucleotides that are arranged in a specific order. This sequence of nucleotides encodes the genetic information necessary for the organism to develop and function. The DNA segments that determine those characteristics of an individual that are inherited from one generation to the next are called genes. Large areas of the DNA, however, do not appear to contain genes. Some of these regions help regulate the activity (i.e., expression) of genes; for other DNA segments the function is still unknown. Nevertheless, these “noncoding” DNA regions can provide important tools for the study of the genome, as described next.

Many variations in the DNA exist among the genes and noncoding DNA regions of different individuals. Such variants of a DNA sequence are called alleles. A DNA region for which several alleles exist is said to be polymorphic—that is, it exists in many forms. The identification of these variants has revolutionized the study of genetics because it allows researchers to study the inheritance of the alleles and to associate specific alleles with the presence of certain diseases. Several types of polymorphisms are commonly used for genetic analyses, including microsatellite markers and single-nucleotide polymorphisms (SNPs). Microsatellite markers are DNA sequences in which short motifs of two, three, or four nucleotides are repeated several times, with the number of repetitions varying from person to person (see figure 1C). Microsatellite markers are typically found in noncoding DNA regions. SNPs consist of the exchange of single nucleotides in the DNA (see figure 1C) and can be found both in coding and noncoding DNA regions. If SNPs occur within a gene or within a DNA region controlling the activity of a gene, they can result in disease. However, many subtle changes in gene sequence—as well as many other DNA markers used in genetic analyses—have no apparent consequence.

## Review of Experimental Genetic Approaches

### Linkage Approaches

To identify as yet unknown genes involved in alcoholism and other disorders, investigators can search the entire genetic material (i.e., genome) by testing for linkage between polymorphic markers and the expression of the disorder or a related behavior. This means that, by studying families with several affected individuals, researchers analyze whether specific alleles of those markers are more commonly found in people with the disorder or behavior than in people without it. Using markers that are evenly spaced across all chromosomes, one can analyze the entire genome using this approach and thereby identify susceptibility genes with no, or only limited, prior knowledge about the mechanisms underlying the disease process.

Studies using this approach initially focused on understanding the genetic mechanisms underlying disorders that are caused by defects in a single gene and for which the inheritance pattern could be clearly specified. These studies involved statistical tests called parametric linkage analyses, which use DNA markers to locate, or map, a disease gene to a particular chromosomal region. Such analyses led to the identification of the genes causing Huntington’s disease, cystic fibrosis, Duchenne muscular dystrophy, and hundreds of other genetic disorders.^1^

Alcoholism, however, is a complex disease, with multiple genetic and environmental factors contributing to the disorder. As a result, it may be impossible to specify a particular inheritance pattern of the disease. In this instance, researchers can use another type of statistical test called nonparametric linkage analysis as a powerful tool to identify genes involved in the disorder. This statistical approach, which does not require a specific model of how a disease is inherited, typically involves identifying families with many affected family members who are thought to have inherited genes that increase the susceptibility to the disease.

**Figure 2:**
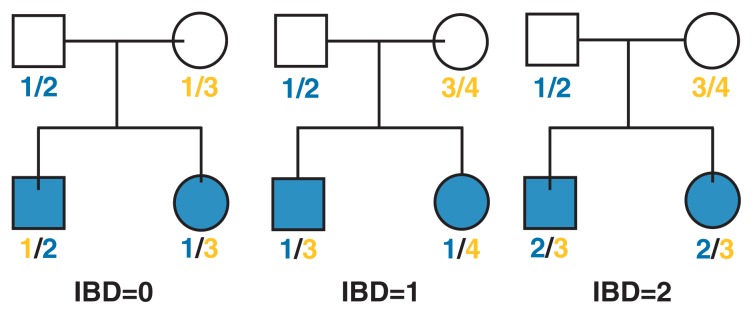
Determination of identity by descent (IBD). For each nuclear family of two siblings and their parents, the illustration shows the genetic makeup of a marker with four variants (i.e., alleles). In the left panel, both siblings have inherited allele 1. However, the brother inherited allele 1 from the mother whereas his sister inherited allele 1 from the father. Therefore, they have no shared alleles (IBD = 0). In the middle panel, both siblings inherited allele 1 from the father (IBD = 1). In the right panel, both siblings inherited allele 2 from their father and allele 3 from their mother (IBD = 2). Siblings who are similar for a trait that is determined by a gene located close to the marker would be expected to share more alleles (i.e., have a higher IBD number) than siblings who are dissimilar for the trait. NOTE: Squares represent males and circles represent females.

All nonparametric linkage analyses are based on a concept called identity by descent (IBD) marker allele sharing (see figure 2). If siblings inherit the same marker allele from the same parent, the allele is called IBD. If the marker being tested is in close physical proximity to a gene influencing the disease or trait under study, then siblings who are similar for the trait would be expected to share more IBD marker alleles. Conversely, siblings who are dissimilar would be expected to exhibit fewer IBD marker alleles near the gene influencing the trait. More recently, nonparametric linkage methods have allowed the inclusion of more extended families beyond sibling pairs in the genetic analysis. Thus, more genetic information can be gained by studying more affected family members. Non-parametric linkage analyses can be used to study both traits that exist in an “either-or” fashion (i.e., qualitative traits, such as having or not having a disease) and traits that vary in severity along a continuum (i.e., quantitative traits, such as quantity or frequency of alcohol use).

### Association Studies and Candidate Genes

Whereas linkage analyses can identify a broad chromosomal region that is likely to contain a gene contributing to a disorder or behavior, association analyses are able to more accurately pinpoint the gene or genes that influence an outcome. One of the most commonly employed experimental designs to identify genes contributing to a disease is that of candidate gene analysis, which seeks to test the association between a particular allele of the candidate gene and a specific outcome.

A candidate gene typically is chosen because it is suspected to play a role in the disorder, either because researchers have some information about the gene’s function that might be related to the disease or because the gene lies in a DNA region that has already been linked to the disorder through linkage studies. In the future, researchers hope to be able to conduct genomewide association studies in which SNPs spaced across the entire genome are tested to identify potential associations with a disease, rather than having to focus on candidate regions or genes. Such an approach, however, is likely to require 50,000 to 300,000 SNPs to test all DNA regions for associations with a disease or trait. The identification of large numbers of SNPs spaced throughout the genome is already under way. In addition, researchers are currently developing new methods to make it feasible for genetic laboratories to determine a person’s DNA sequences (i.e., genotypes) for the large number of markers that will be required in genomewide association studies. Finally, new approaches to the statistical analysis of the vast amount of data resulting from such analyses are being developed, as studying such a large number of markers will create problems with multiple testing and increased potential for false positive results—problems that must be resolved before these strategies are feasible.

#### Population-Based Association Studies

**Figure 3:**
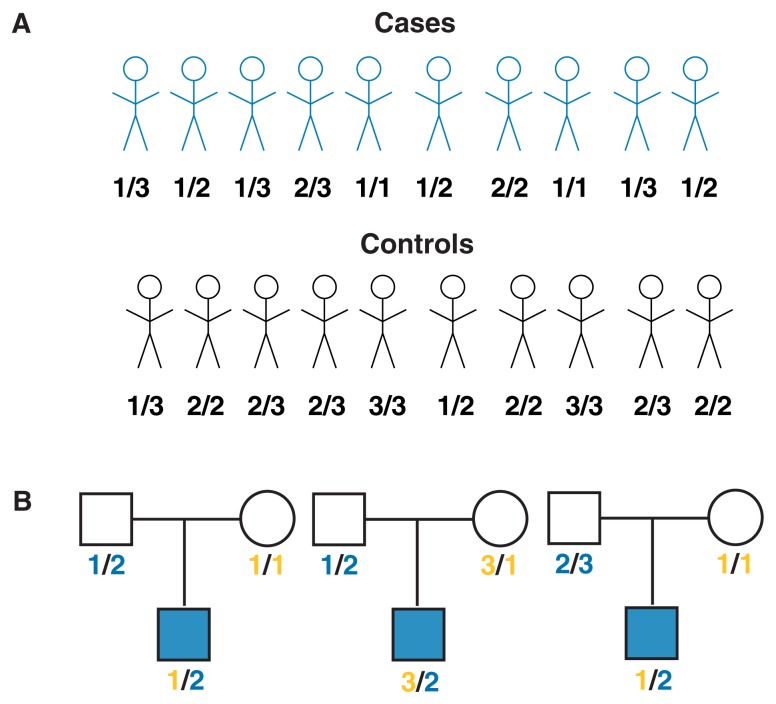
(A) Design of case control studies. These analyses compare the frequencies of alleles in a population of unrelated cases (e.g., alcoholics) and a population of control subjects (e.g., nonalcoholics). In the example shown here, a marker with three different alleles is assessed. The analysis shows that allele 1 occurs in 80 percent of the cases but only 20 percent of the control subjects. This finding suggests that allele 1 may be associated with disease susceptibility (e.g., susceptibility to alcoholism). (B) The transmission disequilibrium test (TDT) tests the hypothesis that a particular marker allele is more frequently transmitted to affected offspring from heterozygous parents. Again, a marker with three alleles is shown. In each of the three trios shown here, the father is heterozygous, carrying allele 2 and either allele 1 or allele 3. The mothers are either homozygous and therefore can transmit only one allele to their offspring (and are thus not informative for the TDT test) or heterozygous. Because in all three cases shown here the affected offspring carry allele 2 from the father, that allele is likely to be associated with the disorder. Information from many trios is tabulated to evaluate the statistical significance of the TDT test. NOTE: Squares represent males and circles represent females.

Many candidate gene studies use population-based association methods—that is, methods that compare the genes of groups of people. For example, in alcoholism research such analyses would involve two samples: a group of alcoholic patients and a control group of nonalcoholic people. Ideally, the two groups would be matched with respect to numerous factors (e.g., age and ethnicity) so that they differ only in disease status. The investigators would then compare the frequencies of various alleles of a marker (e.g., an SNP) within or near the candidate gene (see figure 3A). Evidence of differences in allele frequencies between the two groups is typically interpreted as evidence that the candidate gene contributes to alcoholism susceptibility.^2^

Because of their simplicity, population-based association studies of candidate genes have been widely used and perhaps abused. There are several major problems with this approach in alcoholism research. The first problem is the choice of candidate gene. Numerous biochemical pathways are likely to be involved in addictive behavior, including pathways related to alcohol metabolism. These pathways involve numerous enzymes and other molecules, and the genes encoding all of these molecules are therefore potential candidate genes. Thus, the number of candidate genes is large and increases daily with the application of new technologies to alcohol research. The second problem is that with a disorder that involves multiple genes, such as alcoholism, the effects of each gene are probably small. Therefore, large sample sizes in multiple populations are often required to detect such genes. Third, spurious associations between certain alleles and the disorder are likely to show up if the two samples (i.e., alcoholics and nonalcoholics) are not well matched with respect to important characteristics such as ethnicity.

#### Family-Based Association Studies

To avoid the pitfalls of population-based association studies, Spielman and Ewens (1996) developed a family-based association test—the transmission disequilibrium test (TDT). The primary advantage of the TDT is that it avoids the necessity of including a matched control sample. As originally proposed, the TDT analyzes a nuclear trio consisting of an affected person and his or her parents (see figure 3B). For these three people, researchers then determine the genotype of a marker in or near the candidate gene. If each parent carries two different alleles of the marker gene, then one allele from each parent will be transmitted to the affected offspring and one allele will not be transmitted. Those alleles that have been transmitted from the parents to the affected offspring are considered the “affected” sample, and the remaining alleles are used as “control” sample. Using the information on the transmission of various alleles from many families, investigators can conduct statistical analyses to determine if a particular allele of the marker being tested is associated with disease development. Through the use of such a “within-family” design, the control sample of alleles is perfectly matched to the affected sample of alleles because both samples are transmitted from the same two parents. To expand the use of the TDT, researchers recently have begun to apply it to other designs, such as family-based association tests that include data from both affected and unaffected siblings or from even more extended pedigrees. In addition, the test has been extended to the analysis of quantitative traits in addition to qualitative traits.

### Animal Studies

The use of animal models to study genes related to alcohol use and its consequences may provide important clues that will improve the efficiency of identifying genes underlying human alcohol-seeking behavior. For example, researchers can use animal lines that are well characterized with respect to alcohol-related behaviors to address genetic issues in populations that are genetically more homogeneous, because it is assumed that fewer genes are affecting the behavior of interest. Such an approach should make the isolation of candidate regions and genes faster and more efficient. Animal studies also allow breeding strategies that cannot be performed in humans. Another advantage of animal studies is the high degree of conservation of linked regions of genetic material across many species. This means that genes that are located close to each other on a given chromosome in humans are likely to be also located close to each other on one of the chromosomes in mice, rats, or other animals. As a result, researchers who identify a certain DNA region in mice as playing a role in alcohol-related behaviors can infer the approximate location of the corresponding DNA region on a human chromosome.

Most mouse studies of alcohol preference have used two mouse strains called C57BL/6 (B6) and DBA/2 (D2) that differ greatly in their alcohol preference and, consequently, alcohol consumption. These two strains of mice are inbred, meaning that all animals from that strain are genetically identical and that for each gene, the animals carry two identical alleles (i.e., are homozygous). The consistent difference between the two strains in alcohol preference suggests that these strains carry different alleles at many of the genes influencing alcohol-drinking patterns. These genes are often called quantitative trait loci (QTLs).

Investigators have used a variety of breeding schemes to identify QTLs. For most of these schemes, the first step is to mate animals from the two progenitor lines. The progeny are called F_1_ off-spring. If the progenitors have differing alleles at the relevant QTLs for the trait of interest, then the F_1_ progeny must carry one copy of each allele at all these QTLs (i.e., they are heterozygous for these QTLs). Moreover, all of the F_1_ animals are identical, both in their genetic makeup (i.e., their genotype) and in their observable behaviors or traits (i.e., their phenotype).

**Figure 4:**
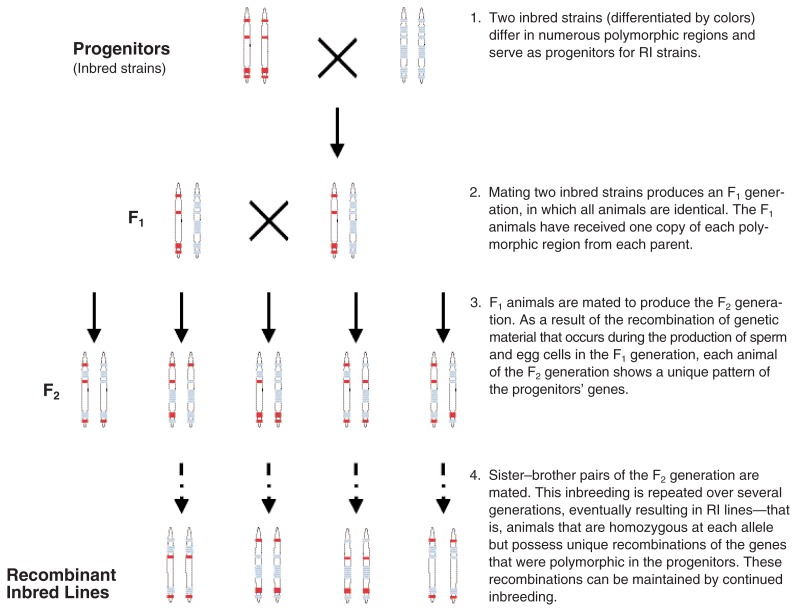
Schematic illustration of the F_2_ breeding design used in genetic analyses, and the generation of recombinant inbred (RI) lines. For the F_2_ design, animals from the F_2_ generation are studied both for the trait under investigation and for their genetic makeup (i.e., genotype). Repeated inbreeding of the F_2_ animals results in the development of multiple RI lines. These lines differ in the composition of the genetic material inherited from the progenitors. However, all animals within an RI line are genetically identical.

Starting with the F_1_ animals, researchers can then use different breeding schemes for subsequent analyses. One approach is the F_2_ design. For this approach, the F_1_ offspring are intercrossed—that is, brothers and sisters are mated—to generate an F_2_ population. In the F_2_ population, the alleles at the QTLs are said to be segregating, meaning that each F_2_ animal has a different combination of alleles at the loci contributing to the trait (see figure 4). This variability arises from the distribution of genetic material from parents to offspring as well as from a process called recombination that occurs during the specialized type of cell division that occurs when the egg and sperm are formed. Recombination is the exchange of genetic material between the two members of a chromosome pair. As a result, each individual of the F_2_ generation has a unique combination of the alleles found in the two progenitor strains. This diversity is reflected in the wide variation in the trait or behavior that is observed in the F_2_ offspring. Therefore, the F_2_ sample is ideal for performing analyses to determine the location of QTLs on the chromosomes (i.e., QTL mapping studies).

Another breeding strategy that is commonly used in inbred mouse studies is the backcross design. In this approach, the F_1_ offspring of two inbred progenitors are mated back to one of the progenitor inbred strains (i.e., are backcrossed), to generate a backcross generation. Compared with an F_2_ sample, the backcross offspring will not be as diverse with respect to their genotype or their phenotype.

An alternative approach that is also commonly used to identify QTLs, particularly using the B6 and D2 progenitors, is the analysis of recombinant inbred (RI) lines. To generate RI lines, brothers and sisters from the F_2_ generation are mated, and this inbreeding is continued for 20 or more generations. As a result of the inbreeding, independent RI lines are created where all animals within an RI line are genetically identical but all RI lines are genetically distinct from one another (see figure 4). Nevertheless, the RI lines are all related to each other because they were derived from the same two progenitors. The RI approach can be a particularly cost-effective means to initially localize QTLs because the genotypes for many RI lines are known and readily available. Thus, an investigator only needs to test the trait(s) of interest in the animals of a set of RI lines and then correlate the trait results with the available genotypic data to identify putative QTLs.

Although the RI approach requires less initial work than the F_2_ approach because sets of RI lines are commercially available, the method is limited by the number of available RI lines. Thus, for a given progenitor cross, typically 20 to 40 RI lines exist, meaning that an investigator has a sample of 20 to 40 different genotypes at a particular locus to assess for the trait of interest. In contrast, the F_2_ approach requires substantially more work, because both the phenotype and genotype must be determined for each animal as each F_2_ animal has a different genotype. The benefit of this approach is that the resulting sample size available for QTL studies is much larger than with RI strains, often in the hundreds or thousands. This large sample size allows for more powerful QTL mapping analyses.

Studies using the B6 and D2 mice have identified QTLs contributing to a variety of alcoholism-related behaviors including alcohol preference, alcohol withdrawal, locomotor activation, and others (Belknap and Atkins 2001; Xu et al. 2002). (For more information on such studies, see the article in this issue by Phillips, pp. 202–207.) Other QTL studies have been conducted in two independently developed rat models of alcohol preference: (1) the alcohol preferring (P) and alcohol nonpreferring (NP) rats and (2) the high alcohol drinking (HAD) and low alcohol drinking (LAD) rats. These analyses have identified additional chromosomal regions that are likely to contain genes influencing alcohol preference.

**Figure 5:**
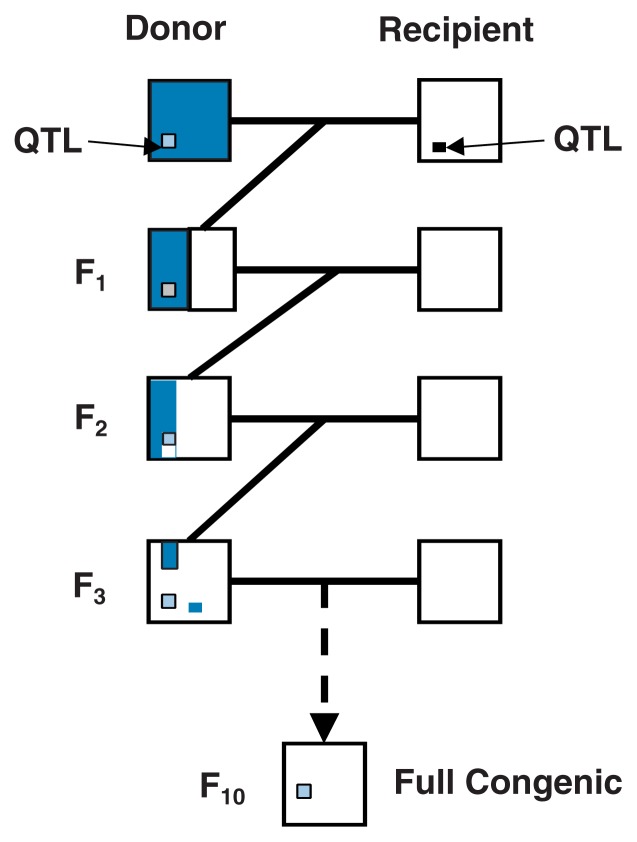
Development of a congenic line in which a quantitative trait locus (QTL) from the donor line is being bred into the recipient line. The blue and white areas represent genetic material inherited from the two progenitors. The F_1_ offspring of a cross between donor and recipient carries approximately 50 percent of the genetic material from each parent. By repeatedly breeding the F_1_ animals and their offspring with the recipient line, the proportion of genetic material from the donor line becomes progressively less until only the QTL under investigation remains from the donor line.

Once QTL regions have been identified, most studies have proceeded to utilize planned breeding combined with analyses of the genotypes of relevant DNA markers in successive generations to develop congenic lines. These are lines of animals in which a DNA region, such as a QTL, is transferred into the genome of another strain through a series of planned matings. One can generate several related congenic lines that carry different or overlapping parts of the original QTL, which allows researchers to substantially narrow down the DNA region that contains the relevant gene(s) (see figure 5).

Another strategy that has been employed in animals to identify genes related to a given trait is to randomly introduce changes (i.e., mutations) into the animals’ genome. In this approach, mutant lines of mice are produced by injecting male animals with a substance called ethyl-nitrosourea (ENU), which introduces DNA mutations throughout the animals’ genome (De Angelis et al. 2000). These males are then mated to normal female mice, and the resulting F_1_ mice are tested with extensive screening protocols to identify those with abnormal traits. Mice with the desired trait are then bred to a known mouse strain for genetic linkage analyses to identify the genetic mutation causing the trait of interest. Because the order of the genes is very similar in mice and humans (and other mammals), the location of a mutation identified in a mouse genome may help predict the location of the corresponding gene in the human genome, suggesting potential candidate genes for related traits in humans. The findings of such analyses can then be validated by studying animals in which the identified gene regions have been inactivated (i.e., knockout animals) or have been introduced artificially (i.e., transgenic animals). (For more information on knockout and transgenic animals, see the article by Phillips, pp. 202–207.)

Thus far, this technique has primarily been used to identify genes that can cause a disease when they alone are mutated (i.e., single-gene mutations). By analyzing such randomly induced mutations on different genetic backgrounds, researchers eventually may be able to also identify genes that only modify the risk for a disease. Although ENU mutagenesis was met with great enthusiasm when it was first introduced, this technique has not yet been highly successful in mapping genes involved in complex traits, such as alcohol-related behaviors.

## From Basic Research to Applied Clinical Settings

The techniques to identify alcohol-related genes described in the preceding sections are still being developed and have yet to realize their full potential and identify genes contributing to alcoholism susceptibility. Nevertheless, clinicians and researchers are already considering the implications of such studies and their findings for alcoholics and their families. These implications range from genetic counseling to gene therapy and pharmacogenetics.

### Genetic Counseling

As challenging as it will be to identify genes involved in alcohol dependence, decisions regarding the use and application of that knowledge will perhaps be even more challenging. How the information regarding genetic susceptibility to alcoholism (and other disorders) will be conveyed to and used by the public is still undetermined. These issues are especially complex because any genes found to be involved in alcoholism are not likely to cause the disease directly but only increase or decrease alcoholism susceptibility. Some of the issues that are likely to face alcohol researchers once they identify susceptibility genes can be illustrated using the example of Alzheimer’s disease (AD) and the apolipoprotein E (*ApoE*) gene. Several alleles of this gene exist, one of which—the *E4* allele—is associated with an increased risk for AD. People carrying at least one *E4* allele have an estimated lifetime risk of AD of approximately 30 percent, whereas the risk of AD is only 9 percent in people who carry no *E4* allele (Post et al. 1997). However, most people who carry an *E4* allele will never manifest the disease, and other people without an *E4* allele can still develop AD. For this reason, screening people for the *ApoE* gene to determine their risk for AD has not been widely accepted in clinical practice, particularly for people who show no symptoms of the disease.

A similar rationale might also apply to any potential genes identified as modifying the risk of alcoholism. However, alcoholism differs from AD in an important way: A person who knows that he or she is at risk for AD cannot take any measures to prevent the disease. People who know that they are at risk for alcoholism because of their genetic predisposition, in contrast, can make informed choices regarding their alcohol consumption and may thereby prevent the disorder. Accordingly, the identification of genes that predispose toward alcohol problems could be beneficial for providing individual-specific risk assessment and could potentially allow some people at higher risk for alcoholism to take preventive measures (i.e., remain abstinent).

### Gene Therapy

Gene therapy is an experimental treatment in which normal genes are introduced into the body’s cells to correct or modify the cell’s function. This technique was first employed successfully in 1990 when it was used to treat severe combined immunodeficiency (SCID), a disease resulting from a deficiency of the enzyme adenosine deaminase (Anderson 1995). This enzyme normally converts a molecule called deoxyadenosine into another molecule called inosine. A person who inherits two defective alleles of the gene responsible for adenosine deaminase production cannot convert deoxyadenosine into inosine. This leads to the rapid accumulation of deoxyadenosine, which is then converted into a toxic product that kills white blood cells involved in fighting infections, resulting in an almost complete failure of the immune system and early death. To treat the disease, researchers removed white blood cells from affected patients, inserted a normal copy of the adenosine deaminase gene into those cells, and reintroduced the modified cells into the body’s circulation. The modified cells then could produce sufficient adenosine deaminase to prevent deoxyadenosine accumulation. This kind of gene therapy is called somatic gene therapy.^3^ It does not cure the disease, however, because the modified blood cells have only a relatively short life span and are continuously replaced by new, defective blood cells. Therefore, the patients require repeated, lifelong treatments.

However, scientists predict that in the future, genetic intervention in the reproductive tissues (i.e., ovaries and testes) may be possible in which the malfunctioning gene will be replaced with a properly functioning gene in the germ cells (i.e., eggs and sperm). This kind of gene therapy is called germline gene therapy. It would ensure that the normal gene, rather than the abnormal gene, is passed on to the offspring. Such an approach will make gene therapy more efficient, because it frees not only the affected person but also his or her progeny from the burden of the genetic disease. Conversely, with current gene therapy one must treat affected people generation after generation. As of the year 2000, more than 300 protocols for gene therapy were submitted for review by the Center for Biologics Evaluation and Research, a division of the U.S. Food and Drug Administration.^4^

The likelihood that gene therapy will be employed to modify the risk of alcohol dependence or other psychiatric disorders is unclear. Gene therapy is currently a complex and inefficient process, even when applied to disorders that result from a defect in a single gene. Gene therapy of disorders influenced by multiple genes as well as environmental factors is even less likely to be an efficient means of disease treatment or prevention.

### Pharmacogenetics

New genetic findings also may have a large impact on the pharmaceutical industry (Roses 2000). For many disorders, treatment with medications is currently a trial-and-error process in which a patient’s physician attempts to determine what particular medicine and dose is most effective for that patient. For example, substantial variability exists in patients’ responses to a given medication, with some patients failing to respond to medicines that are effective in other patients. Similarly, some patients experience severe adverse side effects from medications that are well tolerated by other patients. The new area of pharmacogenetics promises to make this trial-and-error process a thing of the past.

Pharmacogenetics is the application of knowledge about a person’s genetic makeup to predict his or her response to a particular drug. Thus, in the future, physicians may be able to conduct genetic tests before prescribing a medication to determine whether the patient is likely to respond to the medication or to experience adverse side effects. To obtain this knowledge, researchers must compare large numbers of patients who do or do not respond to a medication, or who do or do not experience side effects. They must then analyze the DNA sequences of these differing populations to identify differences that predict the patients’ responses. In this way, a person’s DNA sequence profile can be used to tailor treatment to his or her specific condition, even if the actual DNA sequences that are involved in the disorder are unknown. Some scientists have argued that the discovery of DNA sequence variants relevant to pharmacogenetic issues raises fewer ethical and legal concerns than do genetic analyses that attempt to identify genes associated with a disease. This is because physicians would not test patients for the presence of specific genetic mutations predisposing to a disease, which would be subject to numerous ethical issues. Instead, physicians would test for DNA sequence variants that predispose to certain medical responses (and which may, for example, be related to the person’s metabolism).

## Conclusions

This review has provided an introduction to the tools currently available in the search for genes involved in complex disorders, such as alcoholism, as well as to the issues that likely will be raised by the identification of those genes. This necessarily simplified overview should not convey the impression that the identification of such genes is straightforward. Indeed, many questions remain unanswered, including what study designs are most powerful, what analytic methods are best, and which alcoholism-related traits are most appropriate for analysis. This issue of *Alcohol Research & Health* explores several of these topics. Many more questions will be raised once genes contributing to alcohol dependence are actually identified. However, the identification of such genes can also yield great benefits. Thus, these genes and their gene products may provide the essential data leading to early intervention for people who are at risk for alcoholism because they have inherited susceptibility genes. In addition, enhanced genetic knowledge may lead to the development of more effective treatment of alcoholic patients through a better understanding of the biochemical pathways involved in disease development.

## Footnotes

1 A list of those disorders and genes can be found at the On-line Mendelian Inheritance in Man (OMIM) Web site at http://www3.ncbi.nlm.nih.gov/Omim/.

2 In theory, significant differences in allele frequencies between the alcoholics and nonalcoholics also may be the result of linkage disequilibrium between the candidate gene and the true disease susceptibility gene, which is located within the chromosomal region, but is distinct from the candidate gene. This means that the candidate gene is not actually the causative mutation, but it is so close to the disease-causing mutation that the candidate gene and the disease mutation tend to be transmitted together during the formation of eggs or sperm.

3 Somatic cells are those cells that make up most of the body. Because they are not used for reproduction, like the germ cells, somatic cells and their genetic information are not passed on to the offspring.

4 A list of such protocols is available at http://www.fda.gov/cber/gene.htm.
